# Women in monitoring positions and market risk. Are the stocks of companies with gender diverse boards less volatile?

**DOI:** 10.3389/fpsyg.2022.1049175

**Published:** 2022-11-10

**Authors:** María del Carmen Valls Martínez, Rafael Soriano Román

**Affiliations:** Mediterranean Research Center on Economics and Sustainable Development, Economics and Business Department, University of Almería, Almería, Spain

**Keywords:** gender diversity, board of directors, systematic risk, volatility, stock market risk

## Abstract

Gender equality is included in the United Nations Sustainable Development Goals and in the Global Jobs Pact of the International Labour Organization. Many countries, especially in Europe, are incorporating legal quotas into their legislation to oblige companies to increase the presence of women in the highest positions of responsibility. This measure has been controversial and widely debated, and so it is of great interest to analyze the economic effects that the incorporation of women brings. The aim of this paper is to analyze the relationship between the percentage of women on the board of directors and systematic market risk, measured using the beta of stocks in the S&P 500 and Euro Stoxx 300 indexes from 2015 to 2019. Applying OLS regressions with instrumental variables, fixed effects panel data, and a GMM estimation, the results show a negative and significant relationship for the U.S. market. However, this relationship was not confirmed for the European market.

## Introduction

The board of directors is the main management and control body of a company. It establishes the company’s strategic policies, defines the investment guidelines, determines the intended financing structure, and selects executive personnel (Campbell and Mínguez-Vera, 2008; Ferreira, 2010). The corporate board plays a key role in monitoring the activities of the company and ensuring that managers act in accordance with the interests of shareholders (Chakraborty et al., 2019).

In general, women show less absenteeism at board meetings than men, and so, on gender-diverse boards, absenteeism among men is reduced. As a result, the presence of women leads to more active boards, which increases the monitoring of companies, resulting in a reduction in risk (Adams and Ferreira, 2009). Board gender diversity is also related to a reduction in the reputational risk of a company (Chen et al., 2017), as well as fewer lawsuits (Adhikari et al., 2019), earnings management practices (Kyaw et al., 2015), and information asymmetries (Abad et al., 2017). Companies with a higher percentage of women on the board of directors are less likely to manipulate their financial statements or commit tax fraud (Wahid, 2019), and are more transparent in disclosing their financial risk (Bufarwa et al., 2020).

The board of directors is responsible for the supervision of risk management and the governance of risk-related business decisions (Mcnulty et al., 2013). The board members’ individual risk preferences will affect company decisions. As men are more prone to risk-taking behavior (Birindelli et al., 2020; Plieger et al., 2021), gender diversity on the board will lead to a reduction in corporate risk (Palvia et al., 2020). Indeed, banks suffer fewer defaults when the decision on whether to grant a loan is made by women (Palvia et al., 2015). Moreover, the presence of women on boards is associated with a strong capital structure featuring a larger proportion of long-term financing (Alves et al., 2015), higher stock liquidity (Loukil et al., 2019; Ye et al., 2021), lower stock price crash risk (Qayyum et al., 2021), and reduced stock volatility (Bansak et al., 2011).

Behavioral differences between genders have been extensively studied in psychology, but not sufficiently in corporate finance. If decision-making is influenced by gender, the proportion of women on the board of directors should have an impact on stock value (Huang and Kisgen, 2013). There exists a vast literature on the relationship between board gender diversity and corporate social responsibility (Kyaw et al., 2017; Harjoto and Rossi, 2019; Lu and Herremans, 2019; Al-Qahtani and Elgharbawy, 2020; Valls Martínez et al., 2020a, 2022a). The connection between gender-diverse boards and financial performance, which can be measured based on accounting results (Ekada and Mboya, 2012; Joecks et al., 2013; Ward and Forker, 2017) or market valuation (Campbell and Mínguez-Vera, 2008; Reguera-Alvarado et al., 2017; Valls Martínez and Cruz Rambaud, 2019), has also been extensively analyzed.

However, studies on the influence of board gender diversity on stock market risk are scarce. Moreover, the few existing works on the subject yield contradictory results and are therefore inconclusive (Jizi and Nehme, 2017). The results found may differ in the empirical studies conducted due to the samples used, i.e., the geographical scope and time period analyzed (Adams and Ragunathan, 2017), as well as the methodologies employed. Therefore, more research is needed to really find out how women’s participation on the board of directors influences the volatility of the company’s stocks in the capital markets.

Investors select their portfolios based on two main variables, return and risk, which move in the same direction. Considering two different financial assets, rational investors will always prefer the one with the lower risk when faced with equal returns. They will only be willing to invest in the riskier asset if the expected return is also higher. The total risk of a financial asset is determined by the fluctuation of the market price. Part of this fluctuation is due to the market’s own characteristics, which affect the shares listed on it, although not to the same degree. This is known as systematic risk. Another cause of stock volatility is the specific situation of the company, defined as idiosyncratic risk.

Financial markets are not stable. On the contrary, they are highly sensitive to all events that may affect the economy. At present, they are particularly turbulent because of the COVID-19 pandemic and the Russo-Ukrainian War. According to the literature, women’s risk behavior differs from that of men, and so this paper aims to test whether the percentage of women on boards of directors influences share price stability, and whether companies with more women are more resilient to market fluctuations, resulting in more suitable stocks for risk-averse investors. The object of analysis is systematic risk, as it is the most relevant for the investor and it cannot be eliminated by portfolio diversification, unlike idiosyncratic risk. Indeed, idiosyncratic risk is less significant to the investor, although it is important to other stakeholders (lenders, employees, customers, etc.), who would suffer in the event of losses or bankruptcy of the company (Yang et al., 2019). However, this work is conducted from the point of view of an investor selecting a portfolio.

Based on the extensive literature establishing differences in risk preferences between women and men, it may be expected that a higher presence of women on the board of directors would influence the company’s risk (Eckel and Grossman, 2008; Yang et al., 2019). In heterogeneous groups, it tends to be more difficult to reach consensus, which implies longer decision-making times. Therefore, gender diverse boards could entail more risk. However, board gender diversity implies greater monitoring, which reduces the danger of extreme results and, therefore, entails less risk (Sila et al., 2016).

In general, all investors are averse to risk, but women are considered more risk-averse, leading to more conservative board decisions (Adams and Ragunathan, 2017). Women possess a more in-depth knowledge of the market (Bernardi and Threadgill, 2010), contribute to higher financial performance (Valls Martínez and Cruz Rambaud, 2019), and present greater commitment to corporate social responsibility practices (Valls Martínez et al., 2019, 2022a). In addition, men are more overconfident, which drives them to overestimate expected results and make investments that do not generate value (Huang and Kisgen, 2013). Therefore, the stock volatility of companies with a higher proportion of women on the boards of directors is expected to be lower.

Women, who represent half of the population, are underrepresented on boards of directors, in government and in top positions in general. In OECD countries, women earn, on average, 15% less than their male counterparts (OECD, 2017). One of the main reasons for the lower, and sometimes non-existent, representation of women in management positions is gender stereotyping. Leadership and decision-making skills are more often attributed to men. However, despite widespread stereotypes, women’s participation in top management is increasing, due not only to changes in social values and ethical pressure measures, but also, and mainly, to quotas established by legislation (Johnson and Powell, 1994; Adams, 2016; Birindelli et al., 2020).

The European Commission recommended that its member states develop their legislation to increase the representation of women on boards of directors to at least 40%. As a result, most countries have established mandatory or voluntary legal quotas. In 2020, the percentage of women on corporate boards in countries with mandatory quotas reached an average of 37.6%, while in countries with voluntary quotas it was 24.3%. However, less than 10% of companies had a female CEO. These figures demonstrate the effectiveness of mandatory quotas in achieving gender equality. In the United States, where there is no such nationwide legislation, the percentage of women is barely 22%. Only a few states, such as California, Illinois, Massachusetts, Michigan, New Jersey, Hawaii, and Pennsylvania have proposed a minimum of between one and three women, depending on the size of the board, while Washington advocated a minimum target of 25% by the end of 2022 (World Economic Forum, 2019; Deloitte, 2022).

The purpose of this research is to analyze the relationship between board gender diversity and systematic market risk in Europe and the United States. For this purpose, companies in the Euro Stoxx 300 and S&P 500 indexes for the period 2015–2019 were considered, using OLS regressions, a two-stage regression with instrumental variables, fixed-effects panel data, and the generalized method of moments. The results show that, for the U.S. market but not for the European market, a higher proportion of women on the board of directors is negatively and significantly related to lower systematic risk, measured by stock beta. So far, this relationship appears to have scarcely been explored at all. Indeed, we have only found seven previous works in this regard. Three of these studies analyze the U.S. market, and, while one finds that the relationship is negative and significant (Perryman et al., 2016), two consider that there is no relationship between the two variables (Sila et al., 2016; Peltomäki et al., 2021). Three other studies were conducted on companies in the United Kingdom (Nadeem et al., 2019), Norway (Yang et al., 2019), and Vietnam (Van Vo et al., 2021), and the presence of women on the boards of directors was found to be negatively and significantly related to systematic risk. Finally, a study of Indian banks concluded that there was no relationship between the two variables (Shukla et al., 2021). The different methodologies and periods used in the studies conducted mean that the results are not comparable (Charness and Gneezy, 2012). Hence, the importance of the present research.

This article contributes to the existing literature in the following respects. First, it is the only empirical study analyzing the relationship between stock beta and gender diversity in the European market. Second, it is the most up-to-date analysis in the U.S. market (previous studies ended in 2010, 2012, and 2018). Third, it is the first study to analyze the U.S. and European markets, allowing for a reliable comparison.

The remainder of this article is structured as follows. The second section reviews the literature and establishes the theoretical framework on which to base the study hypotheses. The third section presents the study variables and describes the methodology used in the empirical analysis. The fourth section presents the results of the research. The fifth section shows the discussion of the results. Finally, the last section presents the main conclusions.

## Literature review and theoretical framework

Gender differences in behavior have been extensively studied in fields such as psychology and even experimental economics (Peltomäki et al., 2021). Research prior to the 1980s considered that women possess certain characteristics, such as being more conformist and less aggressive, that lead them to take fewer risks. However, since the 1980s, many studies have argued that there are no gender differences, claiming, for example, that female and male entrepreneurs have similar personalities (Johnson and Powell, 1994). However, most of the literature considers women to be more cautious (Levin et al., 1988) and men to be more prone to risk-taking (Hudgens and Fatkin, 1985; Sila et al., 2016; Li and Yan, 2021).

Studies by psychologists and sociologists have concluded that women and men respond differently to non-financial risks, such as alcohol, drugs, gambling, and environmental damage. In the field of insurance, women have been shown to have fewer traffic accidents and injuries than men (Karacasu and Er, 2011; Singh, 2017). However, the field of financial decisions from a gender perspective has been studied far less (Eckel and Grossman, 2002; Fehr-Duda et al., 2006).

Regarding investments, women behave more conservatively than men (Agnew et al., 2003; Watson and McNaughton, 2007), selecting lower-risk assets (Jianakoplos and Bernasek, 1998; Sundén and Surette, 1998; Charness and Gneezy, 2012). A greater presence of women on the board of directors is associated with lower risk-taking in mergers and acquisitions, as they are better at negotiating lower valuations (Levi et al., 2014). Similarly, investments made by female executives had higher announced returns than those made by their male counterparts (Huang and Kisgen, 2013). Banks with more female directors were also found to invest in less risky positions (Abou-El-Sood, 2019), and the results obtained by mixed-gender groups of market operators were less volatile than those corresponding to all-male groups (Cueva and Rustichini, 2015).

Studies in the field of psychology have shown that men are more overconfident than women when performing more difficult activities, especially if such activities are traditionally considered to be masculine (Barber and Odean, 2001). Therefore, in management and financial decision-making, women are more risk-averse (Berger et al., 2014; Palvia et al., 2015, 2020). A large number of similarities have been found between both sexes in terms of entrepreneurial personality, leadership style, and ability to process and react to information. However, one important difference is men’s greater propensity to take risks, which increases in more uncertain environments where ambiguity is higher (Powell and Ansic, 1997). However, it should not be understood that women’s higher aversion to risk implies that they make suboptimal decisions that lead to the destruction of value in the company (Khan and Vieito, 2013). In fact, for companies with female CEOs, the risk assumed by the company will decrease, and it will have less indebtedness, less volatile profits, and a higher probability of surviving (Elsaid and Ursel, 2011; Palvia et al., 2015; Faccio et al., 2016).

It is important to remember that although the different gender attitudes to risk are significant, the difference is not large. Moreover, when women and men have the same investment knowledge, the differences are attenuated (Dwyer et al., 2002). The reasons for women’s greater risk aversion include their stronger emotional response to the possibility of loss, the greater overconfidence of men, and by how men see uncertainty as a challenge, as opposed to women, who perceive it as a threat (Borghans et al., 2009; Croson and Gneezy, 2009; Baixauli-Soler et al., 2015; Hurley and Choudhary, 2020).

The theoretical framework that explains gender differences in behavior is biosocial, as it integrates social and biological dimensions (Van Staveren, 2014). From the social point of view, gender roles and gender identity are determining factors. Society has ingrained beliefs about the behavior of women and men, and so most of the population is biased in its thinking. Thus, gender-based behavioral differences derive from education, i.e., from the role attributed to one’s gender, and not from the nature of gender itself. In addition, people entrench gender identity by behaving in accordance with the stereotypes attributed to their gender. From a biological perspective, hormones behave differently in women and men in a risk scenario. When above-average benefits are obtained, testosterone levels rise in men, and this may become addictive. A chronic increase in testosterone leads to an increase in impulsivity and more willingness to take risks, since upward market movements are overestimated. In addition, cortisol rises with market volatility, leading to increased anxiety and exaggeration of risk, as downward market movements are overestimated. While cortisol levels are similar in both sexes, in women it produces a greater reaction in the secretion of the hormone oxytocin, which has a calming effect. Thus, women may perform better in contexts of uncertainty and financial stress (Sapienza et al., 2009; Maxfield et al., 2010).

From an empirical point of view, female portfolio managers reduce risk by adopting a strategy of greater investment diversification, which leads them to outperform men, both in normal market conditions and in times of crisis and price volatility, where they show more patience and self-control by holding their investments and trading less frequently. In addition, women show a more cooperative and ethical attitude, while men are more oriented towards protecting their own interests (Van Staveren, 2014).

As women and men have different attitudes to risk, gender diversity in management positions is linked to corporate risk management. Some views claim that male dominance has contributed to financial crises (Adams and Funk, 2012). If women are more risk-averse, better at monitoring activities, and more ethical in their behavior, then they will make less risky decisions in companies, reducing financial risk (Jia, 2019). Hence, the Lehman Sisters hypothesis, according to which the financial problems that occurred at Lehman Brothers could have been avoided if women had been highly involved in the company’s decision-making (Kroes, 2009).

The literature has shown that, within the general population, women are more risk-averse than men. However, is this difference still evident at the managerial level? It is possible that women in senior management behave similarly to men (Schubert et al., 1999; Adams and Ragunathan, 2017; Van Vo et al., 2021). Some studies consider that, at this organizational level, there are no differences in risk propensity or in the quality of decisions (Johnson and Powell, 1994; Lenard et al., 2014). They argue that women who want to be promoted to positions of high corporate responsibility must behave and think like men on a competitive level, and adapt to the prevailing male environment (Adams and Funk, 2012; Adams and Ragunathan, 2017). In other words, the differences between women and men in the general population vanish once women break through the glass ceiling and enter the traditionally male-dominated sphere (Croson and Gneezy, 2009; Sila et al., 2016). In short, the evidence is mixed. Women may be more or less risk-averse than men depending on the context, culture, measure used, sample selected, etc. (Maxfield et al., 2010; Adamus, 2018).

Focusing on market risk and management gender using samples of U.S. companies, Ozdemir and Erkmen (2022) and Peltomäki et al. (2021) found that the presence of a female CEO reduces both total and idiosyncratic risk, but has no relationship with systematic risk. However, Van Vo et al. (2021) found that, in Vietnamese companies, female CEOs are associated with lower systematic and idiosyncratic risk (see Appendix 1).

Studies on companies in the United States (Baixauli-Soler et al., 2015; Hurley and Choudhary, 2020), China (Jebran et al., 2020), South Asia (Yahya et al., 2020), Canada (Chakraborty et al., 2019), Italia (Rossi et al., 2017), and the U.K. (Jizi and Nehme, 2017) found that the percentage of female board members shows a negative relationship with the total risk of the company’s shares in the stock market. Analogous results are found in two studies conducted on a sample of international banks (Birindelli et al., 2020) and a sample of banks in the Arabian Gulf States (Abou-El-Sood, 2019). Moreover, for U.S. companies, Lenard et al. (2014) found that gender-diverse boards were related to lower total and idiosyncratic risk, and Perryman et al. (2016) likewise reported an inverse relationship with total and systematic risk. Yang et al. (2019) established that board gender diversity negatively influences the systematic and idiosyncratic risk of Norwegian companies. In addition, for UK companies, Nadeem et al. (2019) showed that female directors reduced total, systematic, and idiosyncratic risk.

However, studies conducted on U.S. companies concluded that there was no relationship between gender-diverse boards and total risk (Bansak et al., 2011), or total, systematic and idiosyncratic risk (Sila et al., 2016). Similarly, a recent study of Indian banks, Shukla et al. (2021) found no relationship between gender and systematic risk. Loukil et al. (2020), analyzing a sample of French family businesses, reported that female inside directors increase idiosyncratic risk while female independent directors reduce it. Therefore, given that there is no consensus at managerial level and that the number of studies is limited, more research is needed (Hurley and Choudhary, 2020).

The relationship between the presence of women on the board of directors and the company’s risk can be explained by well-established theories, among which the following are the most important.

According to Agency Theory, managers (agents) might make decisions that are for their own benefit but detrimental to the interests of shareholders (principals), since agents and principals do not always have common interests (Jensen and Meckling, 1976; Fama and Jensen, 1983). Board gender diversity strengthens monitoring (Achour, 2022) by restricting opportunities for managers and limiting risky behavior (Jia, 2019; Yahya et al., 2020). Moreover, it reduces the possibility of earnings management practices and manipulation of financial statements (Qayyum et al., 2021). Diverse gender boards increase public and private disclosure of information, which improves transparency and reduces information asymmetries (Jizi and Nehme, 2017; Loukil et al., 2019). In short, the presence of women on the board of directors reduces both conflicts of interest and agency costs, and leads to a reduction in the market risk of the shares (Palvia et al., 2015).

Upper Echelons Theory states that the company’s strategies and policies are established according to the individual preferences of top management, and that these preferences depend on the managers’ values, psychological traits, knowledge, and experience (Hambrick and Mason, 1984). Therefore, the characteristics of the board members, including gender, are determinant in the decision-making processes, in the acquisition of information, and in the handling of contingencies, which all influence performance (Ozdemir and Erkmen, 2022). Consequently, the percentage of women on the board of directors might influence the company’s risk, as women tend to be more cautious and risk-averse, in line with gender-based behavioral differences (Baixauli-Soler et al., 2015; Palvia et al., 2015; Poletti-Hughes and Briano-Turrent, 2019). Thus, as women are more sensitive to risk, they may favor the adoption of less risky business policies (Loukil et al., 2020; Achour, 2022), thereby protecting the shareholders’ interests (Loukil et al., 2019).

According to Human Capital Theory (Becker, 1964), heterogeneous boards, whose members have different personal traits and backgrounds, strengthen the company’s human capital by contributing a broader range of ideas and perspectives. Diversity can increase the quality of corporate governance by reducing opacity, which is especially important for investors in the case of information asymmetries (Loukil et al., 2020). Gender-diverse boards can be a valuable and inimitable resource (Achour, 2022). Moreover, companies need large and stable resources to survive, especially in competitive and complex environments. Based on Resource Dependence Theory (Pfeffer and Salancik, 1978), board members with different characteristics could bring new perspectives and resources that would attract more investors (Jia, 2019). The literature has widely shown that an increased presence of women in top management enhances financial performance, innovation, corporate social responsibility, and company reputation (Valls Martínez and Cruz Rambaud, 2019; Valls Martínez et al., 2022a). Therefore, we may assume that the presence of women in managerial and monitoring positions might help to reduce the company’s market risk.

Stakeholder Theory (Freeman, 1984) establishes that, in order to survive, a company must meet the expectations of not only its shareholders, but also its stakeholders, i.e., customers, suppliers, lenders, employees, governments, and, in general, society as a whole. As women are more empathetic, communicative, sensitive to other people’s problems, and committed to environmental care, etc. (Nguyen and Nguyen, 2015; Gennari, 2018; Sial et al., 2018; Francoeur et al., 2019), they are more likely than men to satisfy the interests of different stakeholders. Therefore, a gender-diverse board of directors may contribute to lowering the company’s risk.

Based on the previous arguments, we predict that a higher presence of women on the board of directors is related to a lower systematic market risk for the company. To test this statement, we formulated the following hypotheses:

*H1*: The percentage of women on the board of directors is negatively related to the market beta of the company’s stocks in the U.S. market.

*H2*: The percentage of women on the board of directors is negatively related to the market beta of the company’s stocks in the European market.

## Methodology

### The dataset

The empirical study was developed with data corresponding to the companies included in the S&P 500 and Euro Stoxx 300 indexes during the period 2015–2019. This sample was selected for two reasons. First, these indexes are representative of the U.S. and European markets, respectively, allowing for reliable comparisons between them. Second, the period is sufficiently broad and current to allow us to draw reliable conclusions. The year 2020 was not included owing to the disruption experienced by the markets due to the COVID-19 pandemic, which would have distorted the results. It would be interesting, in the future, to compare the 5 years prior to the pandemic with the years after. However, companies do not present their annual financial statements until the middle of the following year, and it is later when they are incorporated into the databases. Consequently, at this time it is too premature to perform the study pre-COVID vs. post-COVID era.

The data were obtained from the Bloomberg database, which is frequently used by stock market analysts and portfolio managers. In addition, it has been used in previous scientific work (Nadeem et al., 2019; Valls Martínez et al., 2022c), which supports its reliability and the validity of the results obtained for practical investment management. After eliminating those observations for which any of the variables used were unavailable, the final sample included 1,998 observations for the United States and 1,161 for Europe.

Table 1 shows the composition of the sample by country. As it can be seen, the S&P 500 index is almost entirely composed of companies headquartered in the United States, although 4.55% of its members are companies that have never had their headquarters in the United States or that have moved to other countries. As regards the Euro Stoxx 300 index, France and Germany are the countries with the highest representation, together accounting for 51.91% of the total sample, followed by the Netherlands, Italy, Spain, Finland, and Belgium follow, with a total of 37.62%. Finally, Ireland, Austria, Luxembourg, Portugal, the United Kingdom, and Switzerland account for the remaining 10.47%.

**Table 1:** Sample by countries.

Euro Stoxx 300		S&P 500	
Country	Percent	Country	Percent
France	28.10	United States	95.45
Germany	23.81	Republic of Ireland	1.98
Netherlands	9.59	United Kingdom	1.78
Italy	9.26	Switzerland	0.59
Spain	8.58	Bermuda	0.20
Finland	5.36		
Belgium	4.83		
Republic of Ireland	2.82		
Austria	2.35		
Luxembourg	2.28		
Portugal	1.34		
United Kingdom	1.34		
Switzerland	0.34		

Table 2 shows the average annual values of the dependent and independent variables corresponding to the companies included in both market indexes. It can be seen that the results remain stable during the period analyzed. The size of the board of directors is only slightly larger in Europe than in the United States, with a difference of just one or two members. However, the percentage of female board members is almost 50% higher in Europe, with between 31.80 and 33.03%, than in the United States, with 23%, depending on the year observed. In contrast, the market beta of companies is lower in Europe, indicating higher volatility in the United States.

**Table 2:** Sample description.

Year	Board size	% women on board	Beta
**Section I. U.S. market**			
2015	11.852823	22.453358	1.054897
2016	11.521127	22.652870	1.016576
2017	11.238298	22.643987	1.019949
2018	11.051613	22.712936	0.987257
2019	11.087420	22.949072	1.028492
**Section II. EU market**			
2015	13.783787	32.464087	0.898884
2016	13.530612	33.031053	0.896141
2017	12.694444	32.825930	0.878769
2018	12.541219	31.858068	0.880025
2019	12.613475	31.802274	0.901391

**Figure 1:**
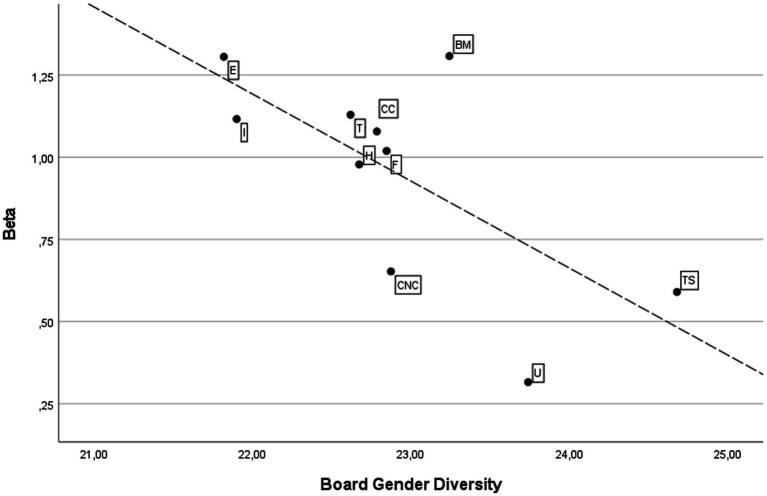
Scatter graph and fitted values by sectors in the U.S. market. B, Basic Materials; CC, Consumer Cyclicals; CNC, Consumer Non-Cyclicals; E, Energy; F, Financials; H, Healthcare; I, Industrials; T, Technology; TS, Telecommunication Services; U, Utilities.

**Figure 2:**
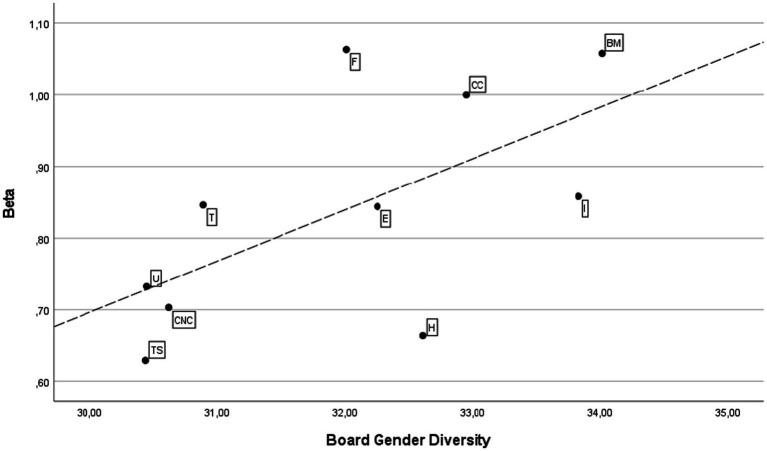
Scatter graph and fitted values by sectors in the EU market. B, Basic Materials; CC, Consumer Cyclicals; CNC, Consumer Non-Cyclicals; E, Energy; F, Financials; H, Healthcare; I, Industrials; T, Technology; TS, Telecommunication Services; U, Utilities.

Figures 1, 2 depict the scatter graph and fitted values by sectors for the dependent and independent variables in the U.S. and European markets, respectively. There is a notable difference between the two markets. The U.S. market shows a negative relationship, so greater gender diversity corresponds to lower volatility. In contrast, the relationship between the variables in Europe is positive.

### Variable description

Table 3 contains the definitions of the variables used in the empirical study, grouped according to the function attributed, as well as the abbreviations to be used hereinafter.

**Table 3:** Variables definition.

	Abbreviation	Variable	Definition
Dependent variable	BETA	Beta	Volatility of a stock against the volatility of the broader market (it is calculated based on trailing five-year prices, on a monthly basis, relative to the S&P 500 or the Euro Stoxx 300 indexes)
Independent variables	BGD	Board Gender Diversity	Percentage of women on board of directors
	BLAU	Blau Index	Blau diversity index
	SHAN	Shannon Index	Shannon diversity index
Control variables	TOQ	Tobin’s Q	Stock price/replacement value
	OPM	Operating Profit Margin	Operating profit to total revenue, as a percent
	SIZE	Company Size	Logarithm of revenue
	INDEB	Indebtedness	Total debt to total equity, as a percent
Instrumental variables	GDP	Policy Board Diversity	Dummy variable, 1 if the company has a board gender diversity policy, and 0 otherwise
	NEBM	Non-Executive Board Members	Percentage of non-executive board members
	IBM	Independent Board Members	Percentage of independent board members
	EMGD	Executive Members’ Gender Diversity	Percentage of female executive members

#### Dependent variable

The stock return, *R*, in the period *t* under consideration, is usually represented in finance as a linear function of market return, as follows:


$$R_{t}=\alpha +\beta R_{Mt}+\varepsilon_{t},t=1,2,\ldots ,T,$$

where *R_Mt_* is the market return for the period *t*. The intercept of the regression, *α*, represents the part of the stock’s return that is independent of the market. The coefficient *β* denotes the degree of intensity with which fluctuations in market return influence stock return. The random disturbance, *ε_t_*, includes the part of the stock’s returns explained by factors independent of the market and dependent on the specific characteristics of the stock (Sharpe, 1963).

The variance of the stock return is given by the following equation:


$$\sigma^{2}\left(R\right)=\beta^{2}\sigma \left(R_{M}\right)+\sigma^{2}\left(\varepsilon \right).$$

The total stock risk is *σ*^2^(*R*), which can be split into two components: *β*^2^*σ*(*R_M_*), which represents systematic or market risk, also known as non-diversifiable risk; and *σ*^2^(*ε*), which represents the own or specific risk, also known as idiosyncratic or diversifiable risk.

The beta parameter, *β*, is known as the volatility coefficient (BETA), and is used as a measure of systematic risk. The higher the beta, the more the asset’s return will increase when the market is rising, but the greater the decline will be when the market is falling. If *β* > 1, the stock moves more sharply than the market and in the same direction. Conversely, if 0 < *β* < 1, the stock is more resilient than the market. Sometimes beta is negative, meaning that stock prices move in the opposite direction to the market, but this is not usual. Risk-averse investors will prefer stocks with *β* < 1, known as defensive betas. On the contrary, risk-prone investors will select stocks with *β* > 1, known as aggressive betas (Valls Martínez et al., 2020b).

The *β*-values were obtained from the Bloomberg database, which calculates them based on the trailing five-year prices, on a monthly basis, relative to the S&P 500 or the Euro Stoxx 300, as applicable. Beta is a proxy for the volatility of a stock against the volatility of the broader market (Yang et al., 2019; Shukla et al., 2021; Van Vo et al., 2021).

#### Independent variables

The independent variable is the percentage of women on the board of directors, which is a proxy for Board Gender Diversity (BGD) in the main management and monitoring body of the company. Only a limited number of papers relate board gender diversity to market risk, and even fewer to systematic risk.

So far, the evidence is mixed. Regarding the U.S. market, Perryman et al. (2016), for a sample of companies from 1992 to 2012 and using OLS regression methodology, found a negative relationship between Beta and Board Gender Diversity. However, Sila et al. (2016), for a sample from 1996 to 2010 and using the generalized method of moments, found no relationship between the two variables, as did Peltomäki et al. (2021), for a sample from 2006 to 2018 and applying the fixed effects and two-stage methodologies with instrumental variables regressions.

In addition, we are aware of three more papers that have identified a negative relationship: Nadeem et al. (2019), on a sample of U.K. firms from 2007 to 2016, using OLS, fixed effects, and two-stage with instrumental variables regressions; Van Vo et al. (2021), considering Vietnamese firms from 2007 to 2015, using OLS and two-stage with instrumental variables regressions; and, Yang et al. (2019), on Norwegian firms from 2003 to 2008, using the Difference-In-Differences regression model. Finally, Shukla et al. (2021) found no relationship between the percentage of women and market beta, in a sample of Indian banks from 2009 to 2016, applying a fixed effects regression model.

To test the robustness of the results and to isolate any causal relationship between BETA and women’s participation, and in line with the literature (Campbell and Mínguez-Vera, 2008; Adams and Ragunathan, 2017; Nadeem et al., 2019; Ozdemir and Erkmen, 2022), the Blau and Shannon diversity indexes, will be used as proxies for gender-diverse boards, as they are considered an optimal approach for quantifying diversity within a group.

The Blau index (BLAU; Blau, 1977) is defined as follows:


$$1-\overset{n}{\underset{i=1}{\sum }}p_{i}^{2}.$$

The number of categories is represented by *n* (in our case, two: women and men) and the percentage of members of each category in the total group by *p*. The index value ranges from 0 (when there is only one gender on the board of directors) to 0.5 (when women account for 50% of the board seats and men for the other 50%).

In turn, the Shannon index (SHAN; Shannon, 1948) is calculated as:


$$-\overset{n}{\underset{i=1}{\sum }}p_{i}\cdot \ln\;\;p_{i}.$$

The meanings of *n* and *p* are the same as in the Blau index, although the Shannon index ranges from 0 (when all board members are of the same gender) to 0.6931 (when women and men account for the same proportion), and is more reactive to small differences in the board composition.

The validity of both indexes for measuring diversity can be observed, as they cannot take negative values, they will be zero when homogeneity is absolute, their value will increase with diversity, and they are upper bounded, as mentioned above (Miller and Triana, 2009).

#### Control variables

It is usual to include accounting and/or market financial performance measures as control variables. The accounting measures represent the current situation of the company derived from its previous trajectory. Market measures, on the other hand, show the company’s long-term future expectations (Valls Martínez et al., 2022a). This study takes the Operating Profit Margin (the ratio of operating profit to total revenue) as the accounting variable (OPM), and Tobin’s Q (the ratio of stock price to replacement value) as the market variable (TOQ).

Two of the control variables used in almost all studies are firm size (defined by total assets, revenues, or number of employees) and level of indebtedness. Accordingly, we have included the logarithm of revenues (SIZE) and the debt-to-equity ratio (INDEB) as proxies for firm size and indebtedness, respectively.

News about the sector to which the firm belongs can affect stock volatility, so dummy variables have been included as control variables to account for the effect of the sector in the empirical analysis. Specifically, firms are grouped into 10 sectors: basic materials, consumer cyclicals, consumer non-cyclicals, energy, financials, healthcare, industrials, technology, telecommunication services, and utilities.

The sign and significance of the relationship between these variables and the firm’s market beta is not uniform among previous empirical studies. For example, indebtedness is often shown to be negatively and significantly related to beta (Baixauli-Soler et al., 2015; Chakraborty et al., 2019; Qayyum et al., 2021; Van Vo et al., 2021), but other studies have shown a positive relationship (Peltomäki et al., 2021; Ozdemir and Erkmen, 2022), or even no relationship (Jizi and Nehme, 2017; Birindelli et al., 2020). The same applies to the other variables.

#### Instrumental variables

To avoid biased and inconsistent regression estimators due to reverse causality and endogeneity problems between BETA and the percentage of women on the board of directors, and in accordance with the literature, one of the methodological procedures employed was a two-stage instrumental variable regression. The instruments must be correlated with the endogenous variable that they replace, i.e., with board gender diversity, but not with the error term in the estimation of beta (Baum et al., 2007). The following variables were used as instruments in the study performed (Valls Martínez and Cruz Rambaud, 2019; Valls Martínez et al., 2022b): (1) a dummy variable based on whether the company applies Gender Diversity Policies on the board (GDP), expecting that, if so, this body will be more gender-diverse; (2) the percentage of Non-Executive Board Members (NEBM), as the more non-executive members there are, the more women could occupy seats on the board; (3) the percentage of Independent Board Members (IBM), since most women on the board tend to belong to the category of independent members; and (4) Executive Member Gender Diversity (EMGD), assuming that, if the company is more inclined to apply gender policies, then these will affect all levels of the organization, from the lowest positions to executive and monitoring positions.

### Methodology

The methodology applied in this study is based on regression models that incorporate various econometric techniques to deal with possible endogeneity problems. For the continuous variables, as is usual before proceeding with the regressions, an analysis of the descriptive statistics and the bivariate Pearson correlations between them is performed. In addition, a test of means and an ANOVA analysis are conducted for the instrumental dummy variable (GDP) in relation to the explanatory variable (BGD).

Indeed, endogeneity is a constant concern in this type of study, since there may be different causes of biased estimators. Variables considered in the analysis, such as company size, could simultaneously influence both the explained and explanatory variables. Larger companies may be at greater risk because they are less agile in adapting to changing circumstances in the economy. In addition, larger companies are more likely to have boards with a larger number of seats and, therefore, may incorporate more women (Adams, 2016). The same could occur with omitted variables that are nonetheless influential. Based on Stakeholder Theory, socially responsible companies enjoy greater legitimacy in the eyes of investors and are perceived as being better managed, which makes them less vulnerable to market fluctuations, thus reducing their risk. Moreover, these companies are more sensitive to ethical issues, so they are more likely to implement equality policies and, consequently, have more women on their boards of directors (Nadeem et al., 2019).

In addition, there could be reverse causality between the dependent and independent variables. On the one hand, if women tend to be more risk-averse, it is logical to think that they will assume less risk in their corporate decisions, and so the market risk of the shares will be lower. On the other hand, companies with lower risk will look for directors with less predisposition for taking risky decisions, possibly leading to greater gender diversity on boards. In addition, women may self-select those companies with lower risk (Sila et al., 2016).

The multivariate empirical analysis starts with an ordinary least squares (OLS) multiple linear regression. Next, we apply one of the most frequently employed techniques for dealing with endogeneity, the use of the lagged dependent variable as a regressor (Francoeur et al., 2019). With the aim of further addressing the problem of reverse causality, and keeping the lagged variable, we next apply a two-stage regression with instrumental variables (Valls Martínez and Cruz Rambaud, 2019). The Sanderson-Windmeijer test (Sanderson and Windmeijer, 2016) and the Anderson test (Anderson and Hsiao, 1981) confirm that the instruments are valid and that there are no identification problems if value of *p* < 0.05. Similarly, with the Sargan test (Sargan, 1958), it must be verified that value of *p* > 0.05. Finally, to eliminate the bias produced by omitted variables, we incorporated the use of panel data treatment with fixed effects to the use of the lagged variable (Adams, 2016). The selection of the fixed effects model over the random effects model is based on the Hausman test (Hausman, 1978). To determine the model that best explains the sample data, in addition to considering the *R*^2^ fit coefficient (higher values are better), the Akaike and Bayesian criteria (lower values are better) are used (Akaike, 1974; Schwarz, 1978).

Furthermore, to test the robustness of the results, we performed additional estimations. First, considering that the use of the lagged dependent variable as a regressor could cause autocorrelation problems, a second-order generalized method of moments (GMM) model was used in order to deal simultaneously with endogeneity and autocorrelation problems (Arellano and Bover, 1995; Yahya et al., 2020). Second, we applied lagged fixed effects using the Blau index as a proxy for gender diversity on the board, with the intention of isolating the causal relationship between this variable and risk (Adams and Ragunathan, 2017). Third, we applied the Shannon index (Campbell and Mínguez-Vera, 2008). Fourth, instead of using the percentage of women on the board of directors as the independent variable, we used the residuals resulting from estimating this variable with the remaining regressors. Fifth, we applied the lagged fixed effects model with board gender diversity as the explanatory variable, but now with all the variables involved winsorized at level 0.01 in order to eliminate extreme values that could distort the results (Valls Martínez et al., 2022b).

The application of these methodologies cannot completely eliminate endogeneity problems, but researchers apply a combination of all of them to test the stability of the results and obtain reliable conclusions (Adams, 2016). Furthermore, from the methodological point of view, the empirical research conducted in this study is in accordance with the literature, as shown in Appendix 1.

## Results

### Descriptive statistics and bivariate relationships

Table 4 shows the descriptive statistics of the continuous variables for the sample companies included in the S&P 500 and Euro Stoxx 300 indexes over the period 2015–2019.

**Table 4:** Descriptive statistics of continuous variables.

Variable	Mean	Median	SD	Minimum	Maximum
**Section I. U.S. market**					
BETA	1.021228	1.023351	0.448188	−0.061792	2.734090
BGD	22.679260	22.22222	8.525444	0	62.500000
BLAU	0.336185	0.345679	0.090373	0	0.500000
SHAN	0.513642	0.529706	0.110126	0	0.693147
TOQ	2.162764	1.578622	2.049475	0	19.365190
OPM	16.774570	15.86832	16.337750	−165.707300	84.602240
SIZE	22.915330	22.86551	1.237210	18.924090	26.966280
INDEB	145.518900	77.57098	341.127100	0	6458.906000
NEBM	86.270310	88.88889	6.791209	60.000000	100.000000
IBM	84.716550	86.66667	8.504586	42.857140	100.000000
EMGD	17.396000	16.66667	12.064190	0	57.142860
**Section II. EU market**					
BETA	0.890904	0.830946	0.366635	0.006099	2.151797
BGD	32.405010	33.333333	11.102540	0	63.636360
BLAU	0.413447	0.444445	0.099103	0	0.500000
SHAN	0.597458	0.636514	0.124603	0	0.693147
TOQ	1.371872	0.975345	1.413182	0	12.998710
OPM	18.929510	11.131230	41.218220	−286.022400	424.890100
SIZE	22.481850	22.497540	1.575104	15.138870	26.1864600
INDEB	109.779600	72.002790	117.396000	0	757.930500
NEBM	89.319900	92.307690	11.218650	40	100.000000
IBM	61.986970	61.538460	25.402800	0	100.000000
EMGD	12.826750	12.500000	10.594130	0	50.000000

With respect to the BETA variable, the median of the distribution of U.S. companies was above 1, while in European companies it was below 1. Specifically, the values were 1.023351 and 0.830946, respectively. This means that while more than half of U.S. companies had aggressive betas, European companies had defensive betas. In other words, the volatility of U.S. companies was higher than that of European companies. In addition, the standard deviation of beta in the S&P 500 was greater than in the Euro Stoxx 300.

The percentage of women on the board of directors (BGD) was, on average, substantially higher in Europe. Indeed, while U.S. companies had less than 23% female board members, European companies were well above the average of 32%. These figures contrast with the percentage of female executive members, where the United States was 4.6% ahead of Europe. It is also noteworthy that the percentage of independent board members in the U.S. market was 22.73% higher than in Europe.

U.S. companies were significantly better valued by the market than European companies (Tobin’s Q was 2.16 in the United States versus 1.37 in Europe). They were also larger and significantly more indebted, showing a narrower operating margin but with a lower standard deviation.

Tables 5, 6 provides the Pearson’s bivariate correlations between the continuous variables. No high correlations were found between the explanatory variables that could give rise to multicollinearity problems.

**Table 5:** Pearson correlations between continuous variables in the U.S. market.

Variable	BETA	BGD	BLAU	SHAN	TOQ	OPM	SIZE	INDEB	NEBM	IBM
BGD	−0.1000^***^ (0.0000)	1.0000								
BLAU	−0.1056^***^ (0.0000)	0.9649^***^ (0.0000)	1.0000							
SHAN	−0.1061^***^ (0.0000)	0.9399^***^ (0.0000)	0.9936^***^ (0.0000)	1.0000						
TOQ	−0.0710^***^ (0.0015)	0.0006 (0.9777)	−0.0013 (0.9535)	−0.0065 (0.7700)	1.0000					
OPM	−0.1433^***^ (0.0000)	−0.0168 (0.4526)	−0.0042 (0.8504)	0.0041 (0.8539)	0.1508^***^ (0.0000)	1.0000				
SIZE	0.0479^**^ (0.0321)	0.0811^***^ (0.0003)	0.0912^***^ (0.0000)	0.0954^***^ (0.0000)	−0.3088^***^ (0.0000)	−0.1405^***^ (0.0000)	1.0000			
INDEB	0.0481^**^ (0.0315)	−0.0172 (0.4416)	−0.0117 (0.6026)	−0.0078 (0.7285)	−0.0290 (0.1956)	0.0023 (0.9171)	0.0380 (0.0895)	1.0000		
NEBM	−0.0211 (0.3449)	0.1471^***^ (0.0000)	0.1649^***^ (0.0000)	0.1695^***^ (0.0000)	−0.0593^***^ (0.0080)	−0.0276 (0.2178)	0.1049^***^ (0.0000)	0.0446^**^ (0.0461)	1.0000	
IBM	−0.0548^**^ (0.0142)	0.1313^***^ (0.0000)	0.1549^***^ (0.0000)	0.1592^***^ (0.0000)	−0.0153 (0.4932)	0.0105 (0.6376)	0.0360 (0.1078)	0.0366 (0.1022)	0.5967^***^ (0.0000)	1.0000
EMGD	−0.0342 (0.1266)	0.2942^***^ (0.0000)	0.2797^***^ (0.0000)	0.2737^***^ (0.0000)	0.0334 (0.1361)	−0.0135 (0.5476)	0.0609^***^ (0.0065)	0.0489^**^ (0.0287)	0.0949^***^ (0.0000)	0.0825^***^ (0.0002)

Value of p in parentheses. ^***^, ^**^ and ^*^ indicate a significance of less than 1%, less than 5% and less than 10%, respectively. Number of observations = 1,998.

**Table 6:** Pearson correlations between continuous variables in the EU market.

Variable	BETA	BGD	BLAU	SHAN	TOQ	OPM	SIZE	INDEB	NEBM	IBM
BGD	0.0708^**^ (0.0159)	1.0000								
BLAU	0.0803^***^ (0.0062)	0.9351^***^ (0.0000)	1.0000							
SHAN	0.0799^***^ (0.0065)	0.8976^***^ (0.0000)	0.9916^***^ (0.0000)	1.0000						
TOQ	−0.2464^***^ (0.0000)	−0.0529^*^ (0.0715)	−0.0691^**^ (0.0185)	−0.0775^***^ (0.0083)	1.0000					
OPM	−0.1936^***^ (0.0000)	−0.0581^**^ (0.0477)	−0.0623^**^ (0.0337)	−0.0656^**^ (0.0254)	−0.0784^***^ (0.0076)	1.0000				
SIZE	0.4253^***^ (0.0000)	0.1089^***^ (0.0003)	0.1173^***^ (0.0001)	0.1191^***^ (0.0000)	−0.4349^***^ (0.0000)	−0.2979^***^ (0.0000)	1.0000			
INDEB	0.0121 (0.6799)	0.0163 (0.5791)	0.0292 (0.3203)	0.0343 (0.2430)	−0.2676^***^ (0.0000)	0.0490^*^ (0.0952)	0.1325^***^ (0.0000)	1.0000		
NEBM	−0.0211 (0.3449)	0.1471^***^ (0.0000)	0.1649^***^ (0.0000)	0.1695^***^ (0.0000)	−0.0593^***^ (0.0080)	−0.0276 (0.2178)	0.1049^***^ (0.0000)	−0.0251 (0.3923)	1.0000	
IBM	0.0746^**^ (0.0110)	0.0280 (0.3399)	0.0911^***^ (0.0019)	0.0921^***^ (0.0017)	0.0560^*^ (0.0563)	−0.008 (0.9793)	0.0381 (0.1947)	−0.0342 (0.2439)	0.0684^**^ (0.0197)	1.0000
EMGD	−0.0206 (0.4825)	0.2127^***^ (0.0000)	0.1826^***^ (0.0000)	0.1754^***^ (0.0000)	−0.0169 (0.5643)	0.0343 (0.2428)	−0.0082 (0.7805)	0.0287 (0.3281)	−0.1376^***^ (0.0000)	−0.0098 (0.7386)

Value of p in parentheses. ^***^, ^**^ and ^*^ indicate a significance of less than 1%, less than 5% and less than 10%, respectively. Number of observations = 1,161.

**Figure 3:**
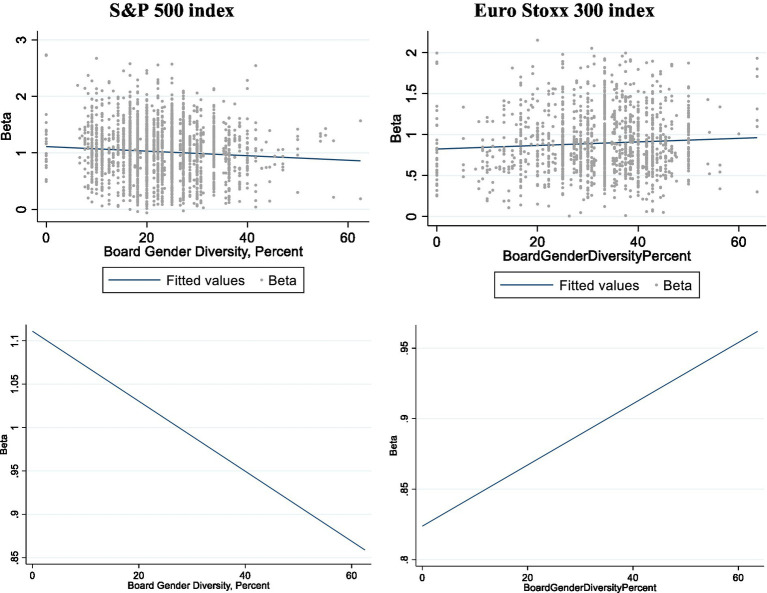
Scatter graph and fitted values Beta – Board Gender Diversity.

The independent variable BGD and the Blau and Shannon indexes showed a significant correlation with BETA, but while the sign of the correlation was negative in the U.S. market, it was positive in Europe. The upper part of Figure 3 depicts the scatter graph and fitted values of BGD and BETA for both samples, where a positive relationship is observed for the U.S. market and a negative one for the European market. However, in the lower part of the graph, where only the adjustment line is represented, the relationships are even more latent, due to the change in the scale of the ordinate axis.

Control variables also showed significant correlations with BETA. The companies better valued by the market and with higher operating margins, corresponded to lower betas. In contrast, larger and more indebted companies presented larger betas. On the other hand, instrumental variables displayed significant correlations with BGD.

The mean test corresponding to the GDP dummy variable, used as an instrument, is presented in Table 7. S&P 500 index companies that implemented board diversity policies had almost 3% more women. However, it is striking that the opposite is true for companies in the Euro Stoxx 300.

**Table 7:** Difference of means in the values of Board Gender Diversity and ANOVA test for the dummy variable of Policy Board Diversity.

	Difference of means test (*t*-test)			ANOVA test	
	Mean group 0	Mean group 1	Difference	*F*	Adjust *R*^2^
*Section I. U.S. market*					
Mean	20.09152	22.98114	−2.889619^***^ (0.0000)	26.37^***^ (0.0000)	0.0105
Percentage	10.60%	89.40%			
*Section II. EU market*					
Mean	35.14651	31.96747	3.179042^***^ (0.0002)	13.94^***^ (0.0000)	0.0089
Percentage	13.56%	86.44%			

Value of p in parentheses. ^***^ indicates a significance of less than 1%.

### Regression analysis

Tables 8, 9 show the results of the regression models applied. First, the OLS estimation, which is the simplest model implemented, shows a negative and significant relationship between BGD and BETA for the S&P 500 companies, at 1% significance level, achieving a fit coefficient of 33.11%. However, for the Euro Stoxx 300 companies, the relationship was positive and not significant, resulting in an *R*^2^ coefficient of 33.73%.

**Table 8:** Regressions in the U.S. market.

Variables	Estimation (OLS)	Lagged estimation (OLS)	IV Lagged estimation (OLS)		Lagged fixed effects estimation
			First-stage IV	Second-stage IV	
Intercept	1.171726^***^ (0.000)	−0.094565 (0.357)	−8.104832 (0.119)	−0.083954 (0.434)	−1.04705^*^ (0.096)
BETA (1 lag)		0.836552^***^ (0.000)	−1.099311^**^ (0.049)	0.833872^***^ (0.000)	0.256033^***^ (0.000)
BGD	−0.003813^***^ (0.000)	−0.002426^***^ (0.000)		−0.004426^**^ (0.011)	−0.001589^***^ (0.004)
TOQ	−0.028652^***^ (0.000)	0.001429 (0.699)	0.154491 (0.197)	0.001795 (0.514)	0.0051151 (0.350)
OPM	−0.001624^**^ (0.020)	−0.000470 (0.260)	−0.020623 (0.124)	−0.000519^*^ (0.094)	0.0002941 (0.415)
SIZE	0.012287 (0.104)	0.014477^***^ (0.001)	0.628315^***^ (0.001)	0.016213^***^ (0.001)	0.0791103^***^ (0.004)
INDEB	0.000107^***^ (0.000)	0.000016 (0.360)	−0.000978^*^ (0.081)	0.000015 (0.234)	−2.16e-06^*^ (0.096)
GDP			2.062570^***^ (0.005)		
NEBM			0.093429^**^ (0.014)		
IBM			0.056573^*^ (0.056)		
EMGD			0.194491^***^ (0.000)		
Sector	Yes	Yes	Yes	Yes	
Adjusted *R*^2^	0.3311	0.8233	0.1155	0.8201	0.8903
*F*-statistic	121.16^***^ (0.0000)	540.24^***^ (0.0000)	12.37^***^ (0.0000)	477.04^***^ (0.0000)	23.57^***^ (0.0000)
Observations	1,998	1,567	1,567	1,567	1,567
Sanderson-Windmeijer test				43.32 (0.0000)	
Anderson test				157.74 (0.0000)	
Sargan test				0.027 (0.9988)	
Hausman test					869.22^***^ (0.0000)
Breush Pagan test					3.466^***^ (0.000)
AIC	1742.140	−773.006		−759.687	−2049.727
BIC	1826.138	−687.295		−673.976	−2012.228

^***^, ^**^ and ^*^ indicate a significance of less than 1%, less than 5% and less than 10%, respectively. Value of p in parentheses. AIC and BIC: smaller is better.

**Table 9:** Regressions in the EU market.

Variables	Estimation (OLS)	Lagged estimation (OLS)	IV Lagged estimation (OLS)		Lagged fixed effects estimation
			First-stage IV	Second-stage IV	
Intercept	−0.865618^***^ (0.000)	0.026168 (0.775)	11.802380 (0.144)	0.064790 (0.472)	−1.459437^**^ (0.032)
BETA (1 lag)		0.852404^***^ (0.000)	−0.000584 (1.000)	0.851097^***^ (0.000)	0.386053^***^ (0.000)
BGD	0.000047 (0.952)	0.000033 (0.929)		−0.000479^***^ (0.002)	−0.000171 (0.690)
TOQ	−0.024566 (0.120)	−0.005458 (0.232)	0.243288 (0.513)	−0.003603 (0.404)	−0.017661 (0.130)
OPM	−0.001498^***^ (0.000)	−0.000331^**^ (0.024)	0.011112 (0.392)	−0.000296^**^ (0.048)	0.000167 (0.636)
SIZE	0.085939^***^ (0.000)	0.006639 (0.116)	0.937557^***^ (0.006)	0.011929^***^ (0.005)	0.088234^***^ (0.004)
INDEB	−0.000014 (0.870)	−0.000083 (0.125)	0.002167 (0.587)	0.000072 (0.121)	0.000122 (0.274)
GDP			−3.376713^***^ (0.001)		
NEBM			−0.000998 (0.975)		
IBM			0.002640 (0.855)		
EMGD			0.226684^***^ (0.000)		
Sector	Yes	Yes	Yes	Yes	
Adjusted *R*^2^	0.3373	0.8548	0.0703	0.8539	0.8916
*F*-statistic	41.35^***^ (0.0000)	269.86^***^ (0.0000)	4.80^***^ (0.0000)	353.69^***^ (0.0000)	25.00^***^ (0.0000)
Observations	1,161	907	907	907	907
Sanderson-Windmeijer test				14.06^***^ (0.0000)	
Anderson test				54.03 (0.0000)	
Sargan test				0.909 (0.8233)	
Hausman test					208.85^***^ (0.0000)
Breush Pagan test					2.49^***^ (0.000)
AIC	249.265	−1205.081		−1052.076	−1788.902
BIC	355.120	−1128.119		−975.132	−1755.231

^***^, ^**^ and ^*^ indicate a significance of less than 1%, less than 5% and less than 10%, respectively. Value of p in parentheses. AIC and BIC: smaller is better.

Second, the lagged dependent variable was used as the explanatory variable, by considering one lag. This lagged OLS regression confirmed the previous relationship between BGD and BETA in both markets, although the model fit improved to 82.33% in the United States and 85.48% in Europe. AIC and BIC criteria confirmed that this model outperforms the first.

Third, a two-stage lagged regression with instrumental variables was applied. For the United States, it is observed that, in the first stage, all the variables used as instruments show a positive and significant relationship with BGD. In the second stage, the negative and significant relationship between the percentage of women on the board of directors and stock beta is again confirmed. In Europe, the sign and significance of the GDP variable is striking, as it indicates that those companies that claim to apply gender diversity policies on the board have, nevertheless, a lower ratio of female directors. In addition, the NEBM and IBM variables were not significant. Regarding the second stage of the regression, it should be emphasized that BGD shows a negative coefficient, significant at the maximum level, contrary to what was indicated by the two previous models. Note that in neither market did the instrumental variables model outperform the lagged OLS regression, as indicated by the *R*^2^ values and the AIC and BIC criteria. Arguably, both models are similar in terms of their validity.

Fourth, panel data with a lagged fixed effects estimation was performed. Once again, for the S&P 500 companies, it was confirmed that an inverse relationship between BGD and BETA was verified at the 0.01 significance level. For Euro Stoxx 300 companies, the relationship was also negative, but not significant. This model achieved the highest *R*^2^ coefficient, with 89.03% for the U.S. market and 89.16% for the European market, and also shows the highest validity according to the AIC and BIC criteria. Therefore, it is the model that best represents the estimation of BETA.

According to the results obtained, H1 is strongly confirmed. We can state that the confirmation of H2 is weak, since it is only confirmed in the model with instrumental variables. Thus, it has been shown that S&P 500 companies with a higher percentage of women on the board of directors have lower market betas, i.e., the stocks exhibit lower volatility and are therefore more suitable for risk-averse investors. However, this relationship is less clear in the European market.

In addition, regarding the remaining variables involved in the study, it is notable that there is a positive and significant relationship between company size and BETA in both markets, such that larger companies show higher volatilities. Thus, risk-averse investors should select smaller companies for their portfolios.

### Robustness checks

The results showed that the best model was the lagged fixed effects regression and, in order to test their robustness, five different strategies were applied. The first of these consisted of a GMM estimation. The next two strategies consisted of the same methodology as the model to be tested, but replacing the independent variable with the Blau and Shannon indexes, respectively, as proxies for gender diversity on the board of directors. In the fourth estimation, the percentage of women on the board was replaced by the residuals resulting from the regression of this variable with the rest of the regressors. Finally, the fifth strategy consisted of winsorizing all the variables to eliminate extreme values that could distort the regression coefficients.

In the U.S. market (see Appendix 2), the greater presence of women on the board of directors was significantly related to lower stock betas. Therefore, H1 was fully confirmed. However, in the European market (see Appendix 3), this negative relationship was not significant, and the GMM model even showed a positive relationship, in line with the results obtained above. Consequently, H2 was not confirmed.

## Discussion

Individual and institutional investors select their portfolios based on expected return and equity risk, both of which move in the same direction. A rational investor will only take on more risk if the expected return increases. Now, will investors be willing to buy a riskier asset or will they prefer a lower-risk asset and settle for a lower expected return? Let us remember that risk implies the possibility of higher profits if the market evolves favorably, but also the possibility of greater losses if it does not. The answer will depend on the investor’s propensity or aversion to risk.

Risk is determined by the volatility of stock prices and is divided into two components: systematic risk and idiosyncratic risk. The former is the most relevant for financial investors, since it cannot be avoided by diversifying their portfolios, unlike the latter. The market beta of the stock indicates how the stock’s return varies with respect to the average return of the market, i.e., the return of the market index, and is the measure of systematic risk. If beta >1, the stock has a higher volatility than the index, and if beta <1, then the stock is more resilient to market fluctuations. When the market is rising, stocks with beta >1 will be of interest for higher returns. However, if the market is down, assets with beta <1 will be more favorable, as the losses will be smaller. Therefore, risk-averse investors will prefer financial assets with lower betas, especially in times of crisis when markets are more volatile (Valls Martínez et al., 2021).

The question is whether there is any relationship between a greater presence of women on the board of directors and stock beta. Traditionally, women have been considered more risk-averse. Therefore, we might wonder whether those boards with a higher presence of women bring about a reduction in beta. Through well-founded and established theories, such as Agency Theory, Upper Echelons Theory, Human Capital Theory, Resource Dependency Theory, and Stakeholder Theory, it can be explained that the presence of women on the board of directors leads to a reduction in the company’s risk (Ozdemir & Erkmen, 2022). Therefore, gender-diverse boards of directors would make decisions more consistent with shareholder interests and curtail potential managerial opportunism (Yahya et al., 2020).

Women tend to find it more difficult than men to hold board positions, as they are usually expected to have more education and experience, and this requirement may be the cause of some of the differences in behavior between the two genders (Adams and Funk, 2012). As a further example of gender discrimination, women who gain access to boards are often not included in financial committees, but are relegated instead to “soft” positions, including monitoring committees (Furlotti et al., 2019). Therefore, it makes sense that more gender-diverse boards are associated with lower risk, as there is greater control.

The greater risk aversion attributed to women is considered one of the main causes of the so-called “glass ceiling,” i.e., the invisible barrier that prevents women from holding senior management positions (Schubert et al., 1999). On the one hand, if women are risk-averse, they may not take risky decisions necessary for the development and growth of the company, to the detriment of shareholders. On the other hand, in complex situations that involve less risk, women are expected to achieve successful results. Furthermore, if women behave similarly to men, they are considered unfeminine and aggressive. In short, the situation for women is unfair because, whatever they do, they will always be judged (Adamus, 2018). Why are women required to behave according to certain standards that are considered irrelevant when men are appointed to the board of directors?

The results of this research show a negative and significant relationship between the percentage of women on the board of directors and stock beta in the U.S. market. Therefore, companies with more women on the board are less volatile and, consequently, more suitable for risk-averse investors. However, this relationship has not been confirmed in the European market. Considering that the period analyzed, 2015–2019, and the methodologies used are coincident for the samples of both markets, we should reflect on what might be the causes of this difference.

The dissimilar results may be due to the fact that the characteristics of U.S. companies differ from those of European companies (Birindelli et al., 2020). Furthermore, the cultural environments are not the same and the economy of each area is specific. In addition, the characteristics of women may vary between countries due to the influence of the institutional and cultural environment (Croson and Gneezy, 2009; Adams and Funk, 2012). It has been shown that the behavioral differences between women and men with respect to risk are more accentuated when the gender gap is greater (Eckel and Grossman, 2002). In 2018, a representative year of the study conducted, the United States was ranked 51st, well behind most European countries. For example, France ranked 12th, Germany 14th, the Netherlands 27th, Spain 29th, and Finland fourth (World Economic Forum, 2018). While it is true that the world is generally moving towards equality, it is not yet a fact. Change is slow, and it is not taking place at the same pace around the world. Professional differences between women and men arise from identity roles, and so the causes must be sought in the motivations and preferences that are created in the social context and not in the competencies, which are the same in both sexes. Thus, from an economic and labor perspective, many differences between women and men disappear in countries with greater gender equality (Adamus, 2018).

As mentioned above, one effect of discrimination is that women in senior corporate positions are relegated to so-called “soft” committees, such as auditing, personnel, corporate social responsibility, and monitoring. Therefore, more women means more control, since monitoring committees are often occupied by women. Consequently, if the gender gap widens in the United States, more women will be relegated to monitoring positions, thereby exercising more control over risk.

In addition, note that corporate beta is, on average, 14.63% higher in the United States than in Europe. This implies that the margin of influence of gender diversity on boards is smaller in Europe, which could also explain part of the difference in results.

It has been argued that, although under normal circumstances there may be no differences in behavior between women and men, gender diversity can be influential in more difficult situations and during periods of crisis, where more innovative ideas are required. Heterogeneous groups bring diversity of thought and, as women are more stakeholder-oriented, they help the company to better address problems and lack of trust (Adams and Ragunathan, 2017). It could be argued that gender diversity helps to generate economic stability (Lenard et al., 2014). Since the more volatile environments are more difficult for companies, the influence of women may be more noticeable in the United States than in Europe.

Few previous studies have related the presence of women on the board of directors to systematic risk, and the results are mixed: four studies considered this relationship to be negative (Perryman et al., 2016; Nadeem et al., 2019; Yang et al., 2019; Van Vo et al., 2021), while three found no relationship (Sila et al., 2016; Peltomäki et al., 2021; Shukla et al., 2021). All refer to a single country and, given that the methodology and variables used are different, they do not allow for a reliable comparison. Hence, the special value of the present investigation. Not only does it add empirical evidence to a little-studied issue, it also makes it possible to compare two important markets in the world economy, those of the United States and Europe.

In summary, the contributions of this article are: (1) it provides empirical evidence that supports the theory on the relationship between the presence of women on the board of directors and the stocks’ market risk; (2) it contributes to the limited research conducted on the U.S. market; (3) it is the first research conducted on the European market; (4) it allows for a comparison of the results obtained in the U.S. and European markets by analyzing the same period and employing the same methodology; (5) by showing the financial benefit of including women on the board of directors, it contributes to the target of the Fifth Sustainable Development Goal of the United Nations: “Ensure women’s full and effective participation and equal opportunities for leadership at all levels of decision-making in political, economic, and public life.”

## Conclusion

This study analyzes the relationship between the proportion of women on the board of directors and systematic stock risk in S&P 500 and Euro Stoxx 300 companies over the period 2015–2019. The results show that the greater presence of women is significantly related to lower risk in the U.S. market. However, this relationship is not fully confirmed in the European market.

From a theoretical perspective, the greater risk aversion of women, which would account for the negative relationship empirically analyzed, is justified based on Agency Theory, Upper Echelons Theory, Human Capital Theory, Resource Dependence Theory and Stakeholder Theory. The possible causes that could lead to the differences found in the study between the U.S. and European markets are the following: (1) the different characteristics of the companies in both markets; (2) the different environments in the United States and Europe that shape the characteristics and competencies of women; (3) the wider gender gap in the United States, which amplifies these differences much more than in Europe; (4) the discrimination suffered by women, who are often relegated to “soft” committees; (5) the higher volatility of the U.S. market, which provides more opportunities to influence a reduction in risk; (6) this greater volatility accentuates the differences between women and men, as the environment is more turbulent.

In the selection of portfolios, one variable that should be considered is the percentage of women on the board of directors. Those companies with more women not only contribute to social equity but also present less risk, at least in the U.S. market, and, in any case, they do not increase risk in the European market. Investing in companies with more women means investing in more stable stocks with less volatility. Board gender composition is a variable that should be incorporated into the financial analysis models of companies.

This research is relevant for companies, which should consider including more women on their boards as a means of reducing market risk. The conducted research is also relevant for individual and institutional investors, who should consider board gender diversity as a key variable when selecting their portfolios, especially if their investment profile is risk-averse.

The incorporation of women into the labor market is increasing. However, there still exists discrimination against women which prevents their access to the best positions (Fauzi et al., 2017; Klinowski, 2019). This study is important not only from a financial point of view, highlighting an important relationship for the selection of investments, but also from a social perspective, as it enhances the value of women’s contribution to the labor market.

The presence of women on boards of directors can reveal the values of society (Shukla et al., 2021), but we must emphasize that the legal establishment of quotas by legislators to contribute to gender equality is not only an example of social justice, but also has important economic connotations.

This analysis does not include data on the age or specific level of education and experience of the women on the company boards, nor on the tasks performed, the decision-making rules, or the group dynamics, which is a limitation. Although this information is difficult to obtain, it could shed light on the results of our study.

Future research should analyze the gender-risk relationship in other markets, such as the Asian market, extend the sample to include non-listed companies, and perform an analysis by sector of activity and country.

## Data availability statement

The data analyzed in this study is subject to the following licenses/restrictions: Data has been extracted from Bloomberg database, which is a private database. Requests to access these datasets should be directed to https://data.bloomberg.com/.

## Author contributions

MCVM: conceptualization, software, methodology, formal analysis, and writing–review and editing. MCVM and RSR: writing–original draft. RSR: data curation. All authors contributed to the article and approved the final manuscript.

## Funding

This work has been funded by PPUENTE2022/006 (from University of Almería, Spain) to the Consolidated Research Group on Ethics, Gender and Sustainability (SEJ-647).

## Conflict of interest

The authors declare that the research was conducted in the absence of any commercial or financial relationships that could be construed as a potential conflict of interest.

## Publisher’s note

All claims expressed in this article are solely those of the authors and do not necessarily represent those of their affiliated organizations, or those of the publisher, the editors and the reviewers. Any product that may be evaluated in this article, or claim that may be made by its manufacturer, is not guaranteed or endorsed by the publisher.
