# Supplementary material for: Women in monitoring positions and market risk. Are the stocks of companies with gender diverse boards less volatile?
Source: Front Psychol. 2022 Nov 10;13:1049175. doi: 10.3389/fpsyg.2022.1049175 (PMC9687104; doi:10.3389/fpsyg.2022.1049175)
Supplement: Supplementary file 1 [file Data_Sheet_1.docx]

**Appendix 1. Summary of the leading literature about gender diversity and market risk**

| **Reference** | **Main research results** | **Sample** | **Methodology** | **Journal** |
| --- | --- | --- | --- | --- |
| (Ozdemir & Erkmen, 2022) | Female CEOs reduce total and idiosyncratic firm risk (volatility of daily stock returns) | Hospitality firms included in the S&P 1500 from 1992 to 2020 | Fixed effects regression, instrumental variables, and generalized method of moments | International Journal of Contemporary Hospitality |
| (Peltomäki et al., 2021) | Female CEOs and CFOs are negatively associated with total and idiosyncratic risk, but are not related to systematic risk (beta) | S&P 1500 from 2006 to 2018 | Fixed effects regression and Two-stage regression with instrumental variables | Journal of Business Finance & Accounting |
| (Vo et al., 2021) | Female CEOs are linked to lower systematic (beta) and idiosyncratic risk | Listed companies in Vietnam from 2007 to 2015 | OLS regression and two-stage regression with instrumental variables | Accounting & Finance |
| (Jebran et al., 2020) | There is a negative relationship between board gender diversity and stock price crash risk | Chinese companies from 2003 to 2015 | OLS regression | Research in International Business and Finance |
| (Shukla et al., 2021) | Women directors do not influence systematic risk (beta) | Indian banks from 2009 to 2016 | Fixed effects regression |  |
| (Hurley & Choudhary, 2020) | The percentage of women on corporate boards is negatively linked to total risk (stock price volatility) | Selected companies included in the S&P 500 index from 2012 to 2016 | Random effects regression | Corporate Governance |
| (Loukil et al., 2020) | Board gender diversity has a significant and positive effect on idiosyncratic risk | French companies included in the SBF 120 index from 2002 to 2012 | Generalized method of moments | Journal in Family Business Management |
| (Yahya et al., 2020) | Board gender diversity reduces equity volatility (total risk measured by GARCH procedure) | South Asian health care companies from 2010 to 2018 | Generalized method of moments | SAGE Open |
| (Birindelli et al., 2020) | Female directors are negatively linked to total risk (stock price volatility) | International banks from 2008 to 2016 | Fixed effects regression | Corporate governance |
| (Yang et al., 2019) | Board gender diversity is negatively linked to total, systematic, and idiosyncratic risk | Norwegian companies from 2003 to 2008 | Difference-in-differences regression | The Leadership Quarterly |
| (Nadeem et al., 2019) | There is a negative relationship between the percentage of female board members and firm risk (total, systematic, and idiosyncratic) | UK listed companies from 2007 to 2016 | OLS regression, fixed effects regression, two-stage regression with instrumental variables, and a Heckman estimation | International Review of Economics and Finance |
| (Chakraborty et al., 2019) | Board gender diversity is negatively and significantly related to total risk | Canadian companies from 2009 to 2014 | OLS regression | Management Decision |
| (Abou-El-Sood, 2019) | The percentage of women on the board of directors is negatively related to total risk (standard deviation of equity returns) | Banks in the Arab Gulf States from 2002 to 2014 | Fixed effects regression | Pacific Accounting Review |
| (Jizi & Nehme, 2017) | Women on corporate boards reduce total risk (stock price volatility) | UK companies included in the FTSE 350 index from 2008 to 2013 | OLS regression and generalized method of moments | Equality, Diversity and Inclusion: An International Journal |
| (Rossi et al., 2017) | The presence of female board members has a negative relationship with risk (standard deviation of return on assets) | Italian listed companies from 2005 to 2013 | Generalized method of moments | Management Decision |
| (Sila et al., 2016) | There is no relationship between board gender diversity and firm risk (total, systematic, and idiosyncratic) | U.S. companies from 1996 to 2010 | Generalized method of moment | Journal of Corporate Finance |
| (Perryman et al., 2016) | Higher gender diversity in top management teams shows lower systematic risk (beta) and total risk (standard deviation of daily returns) | U.S. companies from 1992 to 2012 | OLS regression | Journal of Business Research |
| (Baixauli-Soler et al., 2015) | Gender-diverse boards of directors behave more conservatively with respect to total risk (standard deviation of stock return) than all-male company boards | S&P listed companies from 2006 to 2012 | Generalized method of moment | Journal of Business Research |
| (Lenard et al., 2014) | The percentage of female directors has a negative relationship with idiosyncratic and total risk (standard deviation of daily stock return) | U.S. companies from 2007 to 2011 | OLS regression | Managerial Finance |
| (Bansak et al., 2011) | The percentage of top women executives is not related to volatility (standard deviation of stock market returns) | U.S. financial institutions in 2007 | OLS regression | Applied Economic Letters |

Source: Own elaboration.

**Appendix 2. Robustness checks in the U.S. market**

| **Variables** | **GMM**  **estimation** (†) | **Lagged fixed effects estimation**  **Blau index** | **Lagged fixed effects estimation**  **Shannon index** | **Lagged fixed effects estimation**  **with residuals** () | **Lagged fixed effects estimation winsorized variables** (+) |
| --- | --- | --- | --- | --- | --- |
| Intercept | -1.820119^**^  (0.028) | -0.997713  (0.113) | -0.972131  (0.124) | -1.110588^*^  (0.078) | -0.802602  (0.207) |
| BETA  (1 lag) | 0.619972^***^  (0.000) | 0.256608^***^  (0.000) | 0.256249^***^  (0.000) | 0.255208^***^  (0.000) | 0.254364^***^  (0.000) |
| BGD | -0.001903^**^  (0.018) |  |  | -0.001589^***^  (0.004) | -0.001663^***^  (0.004) |
| BLAU |  | -0.153262^***^  (0.004) |  |  |  |
| SHAN |  |  | -0.118157^***^  (0.006) |  |  |
| TOQ | 0.021101^**^  (0.022) | 0.005043  (0.357) | 0.005068  (0.354) | 0.005223  (0.340) | 0.008094  (0.237) |
| OPM | 0.000611  (0.195) | 0.00300  (0.405) | 0.000302  (0.402) | 0.000371  (0.302) | 0.000454  (0.389) |
| SIZE | 0.950001^***^  (0.009) | 0.077602^***^  (0.005) | 0.076895^***^  (0.005) | 0.080291^***^  (0.003) | 0.068591^**^  (0.013) |
| INDEB | 0.000033^**^  (0.045) | -1.76e-06  (0.902) | -1.56e-06  (0.913) | -2.79e-06  (0.845) | -0.000077^*^  (0.088) |
| Wald Chi2 | 115.12^***^  (0.0000) |  |  |  |  |
| AR(2) | -0.15358  (0.8779) |  |  |  |  |
| Adjusted *R*^2^ |  | 0.8903 | 0.8902 | 0.8903 | 0.8905 |
| *F*-statistic |  | 23.61^***^  (0.0000) | 23.44^***^  (0.0000) | 23.57^***^  (0.0000) | 23.43^***^  (0.0000) |
| Observations | 1567 | 1,567 | 1,567 | 1,567 | 1,567 |
| AIC |  | -2050.074 | -2048.747 | -2049.727 | -2106.618 |
| BIC |  | -2012.576 | -2011.248 | -2012.228 | -2069.119 |

^***^, ^**^ and ^*^ indicate a significance of less than 1 %, less than 5% and less than 10%, respectively.

*p*-value in parentheses.

(†) One-step Arellano–Bover/Blundell–Bond system estimator.

() Determination of residues is not reported for simplicity.

(+) Winsorized variables at the 0.01 level.

**Appendix 3. Robustness checks in the EU market**

| **Variables** | **GMM**  **estimation** (†) | **Lagged fixed effects estimation**  **Blau index** | **Lagged fixed effects estimation**  **Shannon index** | **Lagged fixed effects estimation**  **with residuals** () | **Lagged fixed effects estimation winsorized variables** (+) |
| --- | --- | --- | --- | --- | --- |
| Intercept | 1.297471^**^  (0.033) | -1.447753^**^  (0.033) | -1.440062^**^  (0.034) | -1.452393^**^  (0.033) | -1.457709^**^  (0.032) |
| BETA  (1 lag) | 1.001233^***^  (0.000) | 0.386067^***^  (0.000) | 0.386004^***^  (0.000) | 0.386370^***^  (0.000) | 0.391025^***^  (0.000) |
| BGD | 0.000259  (0.645) |  |  | -0.000171  (0.690) | -0.000058  (0.893) |
| BLAU |  | -0.020484  (0.677) |  |  |  |
| SHAN |  |  | -0.022664  (0.569) |  |  |
| TOQ | -0.023771  (0.109) | -0.017617  (0.132) | -0.017457  (0.135) | -0.017918  (0.124) | -0.020156^*^  (0.084) |
| OPM | 0.000084  (0.774) | 0.000166  (0.636) | 0.000166  (0.636) | 0.000164  (0.640) | 0.000221  (0.682) |
| SIZE | -0.056532^**^  (0.033) | 0.087844^***^  (0.004) | 0.087715^***^  (0.004) | 0.087699^***^  (0.004) | 0.087883^***^  (0.004) |
| INDEB | 0.000129  (0.414) | 0.000122  (0.275) | 0.000123  (0.270) | 0.000118  (0.287) | 0.000135  (0.234) |
| Wald Chi2 | 57.15^***^  (0.0000) |  |  |  |  |
| AR(2) | -3.2447  (0.0012) |  |  |  |  |
| Adjusted *R*^2^ |  | 0.8916 | 0.8916 | 0.8916 | 0.8925 |
| *F*-statistic |  | 25.01^***^  (0.0000) | 25.04^***^  (0.0000) | 25.00^***^  (0.0000) | 25.38^***^  (0.0000) |
| Observations | 907 | 907 | 907 | 907 | 907 |
| AIC |  | -1788.922 | -1789.132 | -1788.902 | -1807.227 |
| BIC |  | -1755.251 | -1755.461 | -1755.231 | -1773.556 |

^***^, ^**^ and ^*^ indicate a significance of less than 1 %, less than 5% and less than 10%, respectively.

*p*-value in parentheses.

(†) One step Arellano–Bover/Blundell–Bond system estimator.

() Determination of residues is not reported for simplicity.

(+) Winsorized variables at the 0.01 level
